# Correction: Shifting Mountains of Electronic Waste

**DOI:** 10.1289/ehp.120-A148-erratum

**Published:** 2012-06-01

The April 2012 Forum article “Shifting Mountains of Electronic Waste” [Environ Health Perspect 120:A148–A149 (2012)] incorrectly suggested that Ruediger Kuehr does not support a ban on e-waste shipments from developed to developing countries. Kuehr does support such a ban in accordance with the 1989 Basel Convention on the Control of Transboundary Movements of Hazardous Wastes and Their Disposal, but he also supports reuse of electronic equipment—which may necessitate international shipments—to reduce the environmental footprint associated with manufacturing.

